# Residual tumor micro-foci and overwhelming regulatory T lymphocyte infiltration are the causes of bladder cancer recurrence

**DOI:** 10.18632/oncotarget.7024

**Published:** 2016-01-25

**Authors:** Alessia Parodi, Paolo Traverso, Francesca Kalli, Giuseppina Conteduca, Samuele Tardito, Monica Curto, Federica Grillo, Luca Mastracci, Cinzia Bernardi, Giorgia Nasi, Francesco Minaglia, Alchiede Simonato, Giorgio Carmignani, Francesca Ferrera, Daniela Fenoglio, Gilberto Filaci

**Affiliations:** ^1^ Centre of Excellence for Biomedical Research, University of Genoa, Genoa, Italy; ^2^ Department of Surgical Sciences and Integrated Diagnostics, University of Genoa, Genoa, Italy; ^3^ IRCCS Azienda Ospedaliero Universitaria San Martino – IST - Istituto Nazionale per la Ricerca sul Cancro, Genoa, Italy; ^4^ Department of Internal Medicine, University of Genoa, Genoa, Italy

**Keywords:** bladder cancer, tumor infiltrating lymphocytes, MAGE, Th1, Th17, Immunology and Microbiology Section, Immune response, Immunity

## Abstract

Bladder cancer has an unexplained, high recurrence rate. Causes of recurrence might include the presence of sporadic tumor micro-foci in the residual urothelial tissue after surgery associated with an inverted ratio between intratumoral effector and regulatory T cell subsets. Hence, surgical specimens of both tumors and autologous, macroscopically/histologically free-of-tumor tissues were collected from 28 and 20 patients affected by bladder or renal cancer, respectively. The frequencies of effector (IFNγ+ and IL17+ T cells) and regulatory (CD4+CD25hiCD127lo and CD8+CD28-CD127loCD39+ Treg) T cell subpopulations among tumor infiltrating lymphocytes were analyzed by immunofluorescence, while the gene expression of MAGE-A1 and MAGE-A2 tumor-associated antigens was studied by RT-PCR. The results show that both the T cell infiltrate and the frequency of MAGE-A1/A2 gene expression were comparable in tumors and in autologous free-of-tumor tissues in bladder cancer, while the autologous free-of-tumor renal tissues showed reduced T cell infiltrate and frequency of MAGE gene expression as compared to the autologous tumors. Importantly, the intra-tumor T effector/Treg cell ratio was consistently <1 in bladder cancer patients (n. 7) who relapsed within two years, while it was always >1 in patients (n. 6) without recurrence (regardless of tumor stage) (*P* = 0.0006, Odds ratio = 195). These unprecedented findings clarify the pathogenic mechanism of bladder cancer recurrence and suggest that microscopically undetectable micro-foci of tumor may predispose to recurrence when associated with an inverted intratumoral T effector/Treg cell ratio.

## INTRODUCTION

Bladder carcinoma is the most common malignancy of the urinary tract and represents the ninth most common cancer worldwide [1]. The main therapeutic options for this disease include transurethral resection for non-muscle-infiltrating stages, and radical cystectomy for more advanced stages [2]. Bladder cancer has an extremely high rate of recurrence. In fact, between 60% and 90% of patients with superficial disease and 50% to 65% of patients with muscle invasive disease relapse after surgery [3, 4, 5, 6]. The reason for this high rate of recurrence, distinctive of bladder cancer, is still unknown. We hypothesized that bladder cancer recurrence may be related to: a) sub-microscopic foci of tumor residual after surgery; b) the intratumoral prevalence of regulatory over effector T cell subsets.

In our study, the T lymphocyte infiltrate and the gene expression of tumor-associated antigens (TAA) were analyzed in specimens from tumoral and macroscopically/histologically free-of-tumor autologous bladder tissues. For comparative purposes, identical analyses were performed on specimens from renal cancer, another genitourinary malignancy that does not present a high recurrence rate [7]. Concerning the T cell infiltrate, its composition (in terms of effector and regulatory T cell subsets) has been related to cancer prognosis [8, 9]. In particular, increased frequency of interferon-gamma (IFNγ) or interleukin (IL)17 positive cells [10, 11] and reduced frequency of T regulatory lymphocytes (Treg) [12, 13] among tumor infiltrating lymphocytes (TIL) were associated with a better prognosis. Hence, IFNγ+ and IL17+ T cells were selected as being representative of effector lymphocyte subsets, while both CD4+ and CD8+ Treg were taken into consideration as regulatory T cell subpopulations. Among the several subsets of CD8+ Treg [14], we concentrated on those phenotypically characterized as CD8+CD28-CD127loCD39hi because frequently present among TIL [15, 16]. The ratio between intratumoral effector and regulatory T cell subsets was correlated with recurrence in a cohort of 13 bladder cancer patients after a two-year follow-up.

With regard to TAA gene expression, two MAGE molecular subtypes (MAGE-A1 and MAGE-A2) were selected for our analyses since their presence has been reported in both bladder and renal cancers [17, 18].

The results of the study showed surprising comparability of both T lymphocyte infiltrate and TAA gene expression pattern between tumoral and macroscopically/histologically free-of-tumor tissues from bladders affected by cancer, while this was not the case for tumoral kidneys. Moreover, they revealed that the recurrence of bladder cancer was invariably associated with a T effector/Treg ratio <1.

## RESULTS

### Circulating effector and regulatory T cell subpopulations in bladder and renal cancer patients

**Figure 1:**
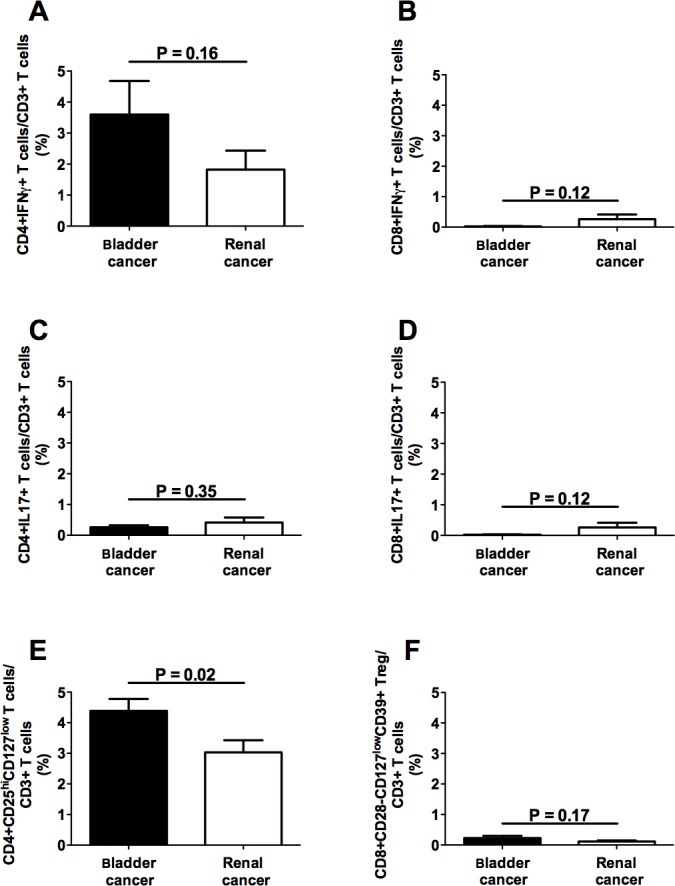
Frequencies of circulating T cell subpopulations in bladder and renal cancer patients Panels A, B, C and D refer to analyses performed on CD4+IFNγ+, CD8+IFNγ+, CD4+IL17+, and CD8+IL17+ T lymphocytes, respectively, comparing the frequencies of each of the above T cell subsets in bladder or renal cancer specimens; panels E and F refer to comparative analyses performed between CD4+CD25hiCD127lo and CD8+CD28-CD127loCD39hi Treg subsets derived from either bladder or renal cancer specimens, respectively; Black bars: analyses performed on cells from bladder cancer patients; open bars: analyses performed on cells from renal cancer patients.

In order to comparatively analyze the relative frequency of effector and regulatory T cell subsets in bladder and renal cancer patients, initial studies were performed on circulating T cells. The frequencies of four effector T cell subsets, namely CD4+IFNγ+, CD8+IFNγ+, CD4+IL17+, CD8+IL17+ T lymphocytes, and two Treg subsets, CD4+CD25hiCD127lo and CD8+CD28-CD127loCD39hi Treg, were comparatively analyzed in the peripheral blood of bladder and renal cancer patients. Figure 1 (panels A-D) shows that no significant differences concerning any of the effector T cell subpopulations were detected in the circulation between patients affected by the two types of tumor. Interestingly, a statistically significant increase in CD4+CD25hiCD127lo Treg frequency was observed among circulating lymphocytes from bladder cancer patients as compared to renal cancer patients (Figure 1, panels E and F). Comparable frequencies of each of the tested T cell subpopulations were observed in patients with TNM >2 and in those with TNM ≤2 (not shown).

### Effector and regulatory T cell infiltrates in bladder and renal cancers

**Figure 2:**
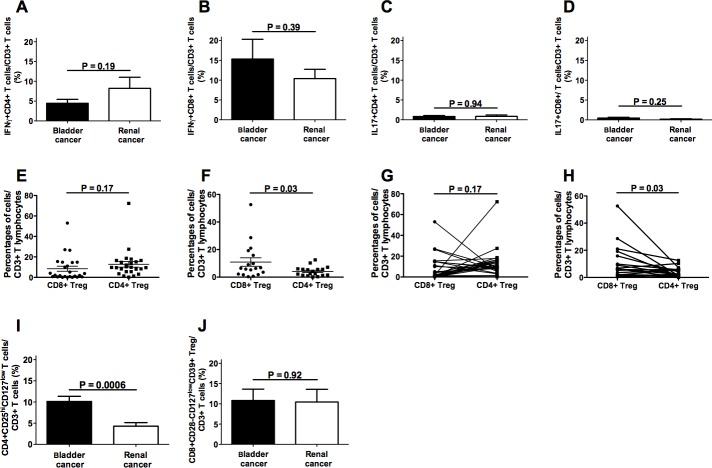
Frequencies of intratumoral T cell subpopulations in bladder and renal cancer patients Panels **A.**, **B.**, **C.** and **D.** refer to analyses performed comparing the frequencies of CD4+IFNγ+, CD8+IFNγ+, CD4+IL17+, or CD8+IL17+ T lymphocytes, respectively, in bladder cancer specimens and renal cancer specimens; panels **E.** and **F.** refer to analyses performed comparing the frequencies of CD4+CD25hiCD127lo and CD8+CD28-CD127loCD39hi Treg subsets in bladder cancer specimens or in renal cancer specimens, respectively; panel **G.** each line associates the frequency values of CD4+CD25hiCD127lo and CD8+CD28-CD127loCD39hi Treg subsets detected in each single bladder cancer specimen; panel **H.** each line associates the frequency values of CD4+CD25hiCD127lo and CD8+CD28-CD127loCD39hi Treg subsets detected in each single renal cancer specimen; panels **I.** and **J.** refer to analyses performed comparing the frequencies of CD4+CD25hiCD127lo or CD8+CD28-CD127loCD39hi Treg subsets, respectively, in bladder cancer specimens and renal cancer specimens.

The absolute counts of intratumoral CD3+ T lymphocytes present in each specimen did not show significant differences between bladder and renal cancer (Supplementary Figure 1). The frequencies of all effector T cell subsets were comparable in the immune infiltrates from specimens of bladder and renal cancers (Figure 2, panels A-D). Interestingly, the frequency of intratumoral CD4+IFNγ+ T cells significantly correlated with that of intratumoral CD8+IFNγ+ T lymphocytes as well as of intratumoral CD4+IL17+ T cells in both types of tumor (Supplementary Figure 2), suggesting their coordinated homing to the tumor site. Comparable frequencies of CD4+ and CD8+ Treg were detected in both types of cancer (Figure 2, panels E and F), although a predominance of either CD4+ Treg or CD8+ Treg was observed in different specimens (Figure 2, panels G and H). Hence, these data indicate that both Treg subsets must be considered in order to achieve a realistic picture of the intratumoral regulatory T cell compartment. Interestingly, a significantly increased frequency of CD4+CD25hiCD127lo Treg was detected among TIL purified from bladder cancer samples as compared to those extracted from renal cancer samples (Figure 2, panels I and J).

### Comparison of effector and regulatory T cell infiltrates between tumor and macroscopically/histologically free-of-tumor tissues

**Figure 3:**
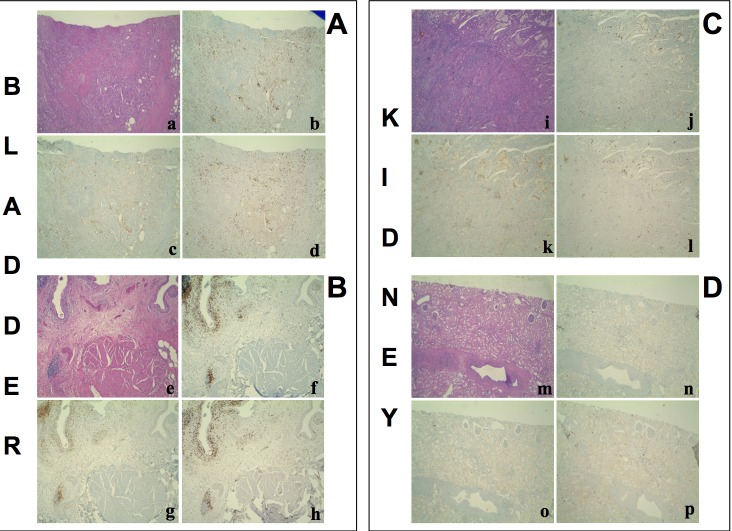
Hematoxylin-Eosin stain and immunohistochemistry for CD3+, CD4+, CD8+ T cells on **A**. a poorly differentiated urothelial carcinoma of the bladder **B.**, the autologous histologically free-of-tumor bladder tissue (both from patient #1), **C**. a papillary type II renal cell carcinoma and **D**. the autologous histologically free-of-tumor cortical renal tissue (both from patient #30). a, e, i, m: Haematoxylin-Eosin stain (4x magnification); b, f, j, n: immunohistochemistry using an anti-CD3 antibody (4x magnification); c, g, k, o: immunohistochemistry using an anti-CD4 antibody (4x magnification); d, h, l, p: immunohistochemistry using an anti-CD8 antibody (4x magnification). a-h: an intense T lymphocyte infiltrate is shown both in the tumour and the histologically free-of-tumor mucosa; lymphocytes are sparse or in nodular aggregates and localize between neoplastic cells as well as in the interstitium. In non-neoplastic mucosa a comparable infiltrate is seen both in the intraepithelial tissue and in the subepithelial connective tissue. i-p: While the tumor shows an evident T lymphocyte infiltrate, rare T lymphocytes are present in the non-neoplastic epithelium.

**Figure 4:**
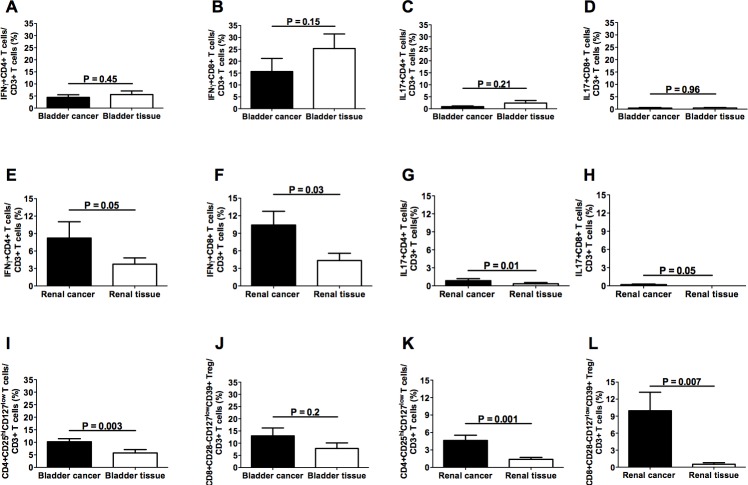
Frequencies of intratumoral T cell subpopulations in bladder cancers and autologous apparently free-of-tumor bladder tissues as well as in renal cancers and autologous apparently free-of-tumor renal tissues Panels A, B, C and D refer to analyses performed on CD4+IFNγ+, CD8+IFNγ+, CD4+IL17+, and CD8+IL17+ T lymphocytes, respectively, from bladder specimens; panels E, F, G, and H refer to analyses performed on CD4+IFNγ+, CD8+IFNγ+, CD4+IL17+, and CD8+IL17+ T lymphocytes, respectively, from renal specimens; panels I and J refer to analyses performed on CD4+CD25hiCD127lo and CD8+CD28-CD127loCD39hi Treg subsets, respectively, from bladder specimens; panels K and L refer to analyses performed on CD4+CD25hiCD127lo and CD8+CD28-CD127loCD39hi Treg subsets, respectively, from renal specimens. Black bars: analyses performed on cells from tumor specimens; open bars: analyses performed on cells from apparently free-of-tumor tissues.

In order to compare the T cell infiltrates present in the tumor with those of the autologous apparently free-of-tumor tissue, surgical specimens were collected from areas of the affected organ that macroscopically appeared free of disease. In parallel, the same areas of apparently normal tissues were histologically analyzed and no signs of tumor infiltration were detected (Figure 3, subpanels a, e, i, m). Preliminary analysis of the absolute cell counts showed comparable values of infiltrating CD3+ T cells in bladder cancers and in the apparently free-of-tumor bladder tissues, while significantly lower amounts of CD3+ T cells were observed in the apparently unaffected renal tissues with respect to renal cancers (Supplementary Figure 1). Importantly, the frequencies of all the effector T cell subsets were comparable in the tumoral and in the corresponding apparently free-of-tumor bladder tissues (Figure 4, panels A-D), while significantly lower frequencies of effector T cell subsets were found in the unaffected kidney tissue as compared to the corresponding, autologous renal cancer (Figure 4, panels E-H). Accordingly, the immunohistochemical staining with anti-CD3, CD4 and CD8 mAbs showed remarkable T cell infiltration in bladder and renal cancers as well as in the apparently free-of-tumor bladder tissue, while a rare T cell infiltrate was present in the apparently free-of-tumor kidney tissue (Figure 3).

Low amounts (<1% of total CD3+ T cells) of CD4+IFNγ+IL17+ and/or CD8+IFNγ+IL17+ T cell subsets were observed in 8 out of 18 tested bladder cancer patients and in 5 out of 17 tested renal cancer patients: no significant differences between tumoral and apparently free-of-tumor tissues, as well as between bladder and renal tissues were observed (not shown).

A decreased frequency of CD4+CD25hiCD127lo Treg was present in the apparently free-of-tumor bladder tissue with respect to the autologous bladder cancer (Figure 4, panels I and J). Similarly to the effector T cell subsets, the frequencies of both CD4+CD25hiCD127lo and CD8+CD28-CD127loCD39hi Treg were significantly lower in the apparently unaffected renal tissue than in the autologous renal cancer tissue (Figure 4, panels K and L).

### MAGE-A1 and MAGE-A2 gene expression in tumor and autologous macroscopically/histologically free-of-tumor tissues

**Figure 5:**
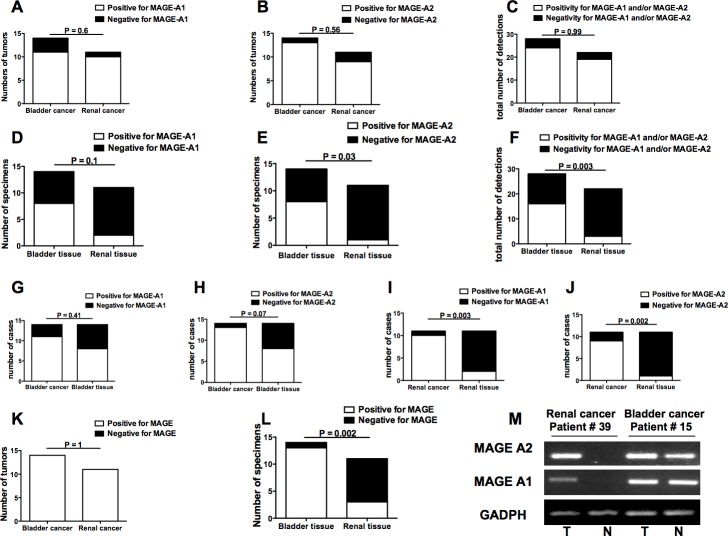
Frequencies of MAGE-A1 and MAGE-A2 gene expression in bladder and renal cancers as well as in bladder and renal apparently free-of-tumor tissues **A.** frequency of bladder or renal tumor showing or not MAGE-A1 gene expression; **B.** frequency of bladder or renal tumors showing or not MAGE-A2 gene expression; **C.** frequency of detections on bladder or renal tumors that resulted positive or not for any MAGE gene expression; **D.** frequency of bladder or renal apparently free-of-tumor tissue showing or not MAGE-A1 gene expression; **E.** frequency of bladder or renal apparently free-of-tumor tissue showing or not MAGE-A2 gene expression; **F.** frequency of detections on bladder or renal apparently free-of-tumor tissue that resulted positive or not for any MAGE gene expression; **G.** comparisons of frequencies of tissue samples showing MAGE-A1 gene expression between bladder cancers and autologous apparently free-of-tumor bladder tissues; **H.** comparisons of frequencies of tissue samples showing MAGE-A2 gene expression between bladder cancers and autologous apparently free-of-tumor bladder tissues; **I.** comparisons of frequencies of tissue samples showing MAGE-A1 gene expression between renal cancers and autologous apparently free-of-tumor renal tissues; **J.** comparisons of frequencies of tissue samples showing MAGE-A2 gene expression between renal cancers and autologous apparently free-of-tumor renal tissues; **K.** frequencies of bladder or renal tumors showing positivity or not for at least one of the two MAGE molecule gene expression; L: frequencies of apparently free-of-tumor bladder or renal tissues showing positivity or not for at least one of the two MAGE molecule gene expressions; **M.** Representative MAGE antigen gene expression analyses performed by RT-PCR on specimens from bladder cancer patient #15 and renal cancer patient #39. The figure shows the amplicons obtained after MAGE-A1, MAGE-A2, and GADPH (as positive control) cDNA amplification in tumors (T) and apparently free-of-tumor tissues (N) run on agarose gel. Panels A-L: Open bars indicate presence of MAGE antigen gene expression while black bars indicate absence of MAGE antigen gene expression.

In order to reveal micro-foci of tumor localization at the molecular level, the expression of two TAA genes, MAGE-A1 and MAGE-A2, which may be expressed by both bladder and renal cancers [17, 18], was comparatively analyzed in tumoral and autologous apparently free-of-tumor bladder and kidney tissues. The frequencies of gene expression of the two TAA genes were comparable in bladder and renal cancers (Figure 5, panels A-C). Conversely, higher frequencies of MAGE-A1 and MAGE-A2 expression were observed in the bladder with respect to the renal apparently free-of-tumor tissue (Figure 5, panels D-F). Accordingly, significant differences in MAGE-A1 and MAGE-A2 gene expression between the tumor and the apparently free-of-tumor tissue were only observed for renal but not bladder cancer (Figure 5, panels G-J). Interestingly, while expression of at least one of the two MAGE genes was detected in 100% of bladder and renal cancers (Figure 5, panel K), MAGE gene expression was a common feature for the apparently free-of-tumor bladder tissue (13 out of 14 positive cases) but not for the apparently free-of-tumor renal tissue (3 out of 11 positive cases) (Figure 5, panel L).

### T effector/Treg cell ratio in the tumor infiltrates of relapsing or non-relapsing bladder cancer patients

**Figure 6:**
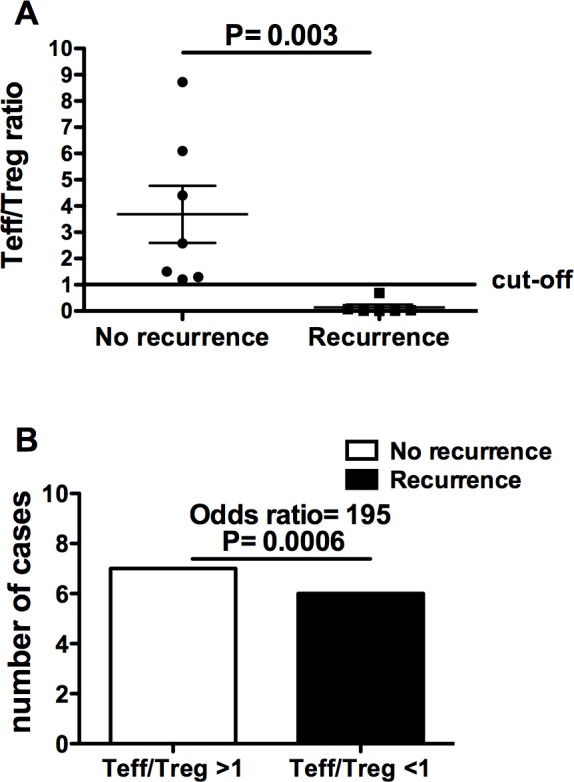
T effector/Treg cell ratio in bladder cancer patients with and without recurrence two years after the surgery **A.** Comparison between the T effector/Treg cell ratio of patients with recurrence and that of patients without recurrence; **B.** contingency analysis on the frequencies of recurrence or non-recurrence in patients with T effector/Treg cell ratios either >1 or < 1.

The observation of comparable T cell infiltrates and TAA gene expression pattern in bladder cancer and in autologous apparently free-of-tumor tissue suggests that sub-microscopic foci of tumor cells are sparse in the urothelium of tumoral bladders, predisposing to recurrence. However, this finding does not explain why not all bladder cancer patients relapse. Keeping in mind the possible role that the intratumoral T cell infiltrate may play in tumor recurrence, we comparatively analyzed its composition in samples taken from 13 patients who relapsed (n. 7) or not (n. 6) within two years from surgery. To this aim, the ratio between the sum of frequencies of the two effector T cell subsets and that of the two Treg subpopulations was calculated for each patient. As shown in Table 1 and in Figure 6, the intratumoral T effector/Treg cell ratio was consistently <1 in patients who relapsed, while it was always >1 in patients who did not relapse, regardless of tumor stage. Interestingly, neither the frequency nor the absolute number of total Treg differed significantly between patients with or without relapse (not shown), underlying the need of taking into consideration the ratio between effector and regulatory T cell subpopulations (and not just single populations) in order to achieve a reliable prognostic marker.

**Table 1:** Calculation of the Teffector/Treg cell ratio in bladder cancer patients affected or not by recurrence within two years from surgery

Patient #	Intratumoral effector T cell subsets				Intratumoral regulatory T subsets		T effector/Treg cell ratio
	CD4+IFNΦ+	CD8+IFNΦ+	CD4+IL17+	CD8+IL17+	CD4+ Treg	CD8+ Treg	
10	7.7*	1.4	0.9	0	10	4	10/14= **0.7**
14	0.2	0.3	0	0	9.8	27	0.5/36.8= **0.01**
15	0	0	0.7	0.3	13.6	0.4	1/14= **0.07**
16	0	0.6	0	0.3	8.2	14.8	0.9/23= **0.039**
18	0.1	0	0	0.1	53	5.6	0.2/58.6= **0.003**
21	0	0.1	0.1	0	10.2	0.1	0.2/10.3= **0.019**
8	6.6	14	0.8	0.3	1.3	1.2	21.7/2.5= **8.6**
11	13.2	62.2	3.2	2.2	3.5	14.8	80.8/18.3= **4.4**
12	7.2	38.9	1.8	0	9.9	8.3	47.9/18.2= **2.6**
13	11.2	65.5	2	2.4	1.9	9.7	81.4/11.6= **7**
17	4.9	29.5	3	0	15.5	17.8	37.4/33.3= **1.1**
19	6.6	16	0	0	2.1	15.2	22.6/17.3= **1.3**
22	8.1	40.2	0	0	26.5	5.6	48.3/32.1= **1.5**

* Data are expressed as percentages of CD3+ T lymphocytes

## DISCUSSION

The results of the present study show that: a) the frequencies of effector T lymphocyte subpopulations, both in the circulation and in tumor infiltrates, are comparable in patients affected by bladder and renal cancers; b) CD4+ Treg frequency is higher in bladder cancer patients than in renal cancer patients, both in the circulation and in tumor infiltrates; c) the macroscopically/histologically free-of-tumor bladder tissue peculiarly presents a T lymphocyte infiltrate whose composition is comparable to what is observed in autologous bladder cancer; d) the apparently free-of-tumor bladder tissue peculiarly shows a pattern of TAA gene expression comparable to that of the autologous bladder cancer; e) the intratumoral T effector/Treg cell ratio in bladder cancer patients with disease recurrence is invariably <1, while it is always >1 in patients without recurrence.

Although considerable progress has been made on bladder cancer pathogenesis [19-22], the high rate of bladder cancer recurrence is still unexplained [23, 24]. Cancer recurrence is likely related to tumor immune escape, a process that is at least partially dependent on an imbalance between effector and regulatory T cell function [25]. Accordingly, cancer prognosis has been related to the degree of tumor infiltration by effector and/or regulatory T cells [26, 27]. These observations suggest that the tumor could influence the T cell infiltrate composition. Subsequently, the identification in an apparently free-of-tumor tissue of a T cell infiltrate resembling, in terms of T lymphocyte subset composition, what is present in the autologous tumor may indicate the presence of not yet histologically detectable foci of tumor cells (an event that in bladder cancer may be due to a genetic predisposition of the transitional epithelium [28] and/or to the early spreading of sub-microscopic metastases within the transitional epithelium). We considered the analysis of the T cell infiltrate as an indirect tool to reveal the presence of sub-microscopic foci of tumors in the apparently free-of-tumor tissue from bladders affected by tumor. For comparative purposes, the same analyses were performed on specimens from kidneys affected by renal cancer. Interestingly, the frequencies of effector (considered as IFNγ+ and IL17+) T cell subpopulations were comparable in the two tumors: this likely rules out the possibility that a different degree (or quality) of the anti-tumor immune response might be responsible for the high recurrence rate that is distinctive of bladder cancer alone. Instead, the frequency of CD4+ Treg was lower in renal cancer compared to bladder cancer indicating that the level of tumor infiltration by Treg may have pathogenic relevance. Since CD4+ Treg frequency was found to be increased both in the circulation and at the tumor site in bladder cancer patients, as compared to what was observed in renal cancer patients, it is likely that both the mechanisms of CD4+ Treg in situ generation (at the tumor site) and recruitment from the periphery could be at play in bladder cancer patients.

The fact that amount and composition of the T cell infiltrates were comparable in the tumor mass and in the apparently free-of-tumor bladder tissue suggests that similar antigenic patterns were present at both sites. The exception constituted by CD4+ Treg, whose intratumoral frequency was greater than that of the apparently free-of-tumor tissue, may reflect the need for a critical size in order the tumor could efficiently generate/recruit Treg. The T cell infiltrate in renal cancer differed between the tumor and the histologically free-of-tumor autologous renal tissue. These findings indicate that the presence of a comparable T cell infiltrate in both the apparently non-tumoral tissue of bladders affected by tumor and in the tumor itself is a specific prerogative of bladder cancer, likely related to the presence of sub-microscopic foci of tumor cells.

In order to molecularly reveal these sub-microscopic tumor foci, we comparatively searched for the expression of genes coding for TAA, such as MAGE-A1 and MAGE-A2 [17, 18] in the tumors and in the autologous free-of-tumor bladder or renal tissues. While the pattern of TAA gene expression in the macroscopically/histologically free-of-tumor bladder tissue was comparable to that of the autologous tumor mass, this was not the case for the un-diseased renal tissue. This further supports the presence of tumor cells in the context of the apparently non-tumoral bladder, but not renal, tissue.

These results do not yet explain why only a certain percentage of patients relapse. Importantly, we observed that only patients with an inverted T effector/Treg cell ratio relapsed. This likely suggests that the multi-focal feature of bladder cancer may result in recurrence only when there is a local impairment of anti-tumor immunity due to overwhelming immunoregulation. Moreover, this unprecedented finding proposes the intratumoral T effector/Treg cell ratio as a new, sensitive biomarker of bladder cancer recurrence that, if validated in a broader series, could lead to a revision of both prognostic and therapeutic standards of bladder cancer.

## MATERIALS AND METHODS

### Subjects

Twenty-eight patients affected by bladder cancer and 20 patients affected by renal cancer were enrolled at the Urology Clinic of the IRCCS – AOU San Martino – IST hospital of Genoa (Table 2). None of the bladder cancer patients had been treated with BCG at the time of enrollment. We collected a heparinized peripheral blood sample before surgery, a surgical specimen from the tumor mass, and a surgical specimen from an autologous area of macroscopically free-of-tumor bladder or renal tissue from each patient. A portion of the specimen from the macroscopically free-of-tumor tissue area was separated from the part on which immunological and molecular tests were performed, and was analyzed histologically.

**Table 2:** Pathological features of patients enrolled in the study

Patient #	Tumor histology	Grading	TNM
1	Bladder carcinoma	2	1a
2	Bladder carcinoma	3	1
3	Bladder carcinoma	3	3a
4	Bladder carcinoma	3	Tis
5	Bladder carcinoma	3	3
6	Bladder carcinoma	2	2
7	Bladder carcinoma	3	Tis
8	Bladder carcinoma	3	2
9	Bladder carcinoma	3	2
10	Bladder carcinoma	3	3
11	Bladder carcinoma	3	3b
12	Bladder carcinoma	3	Tis
13	Bladder carcinoma	3	1
14	Bladder carcinoma	3	3a
15	Bladder carcinoma	3	3b
16	Bladder carcinoma	3	3b
17	Bladder carcinoma	2	Ta
18	Bladder carcinoma	3	3
19	Bladder carcinoma	3	3b
20	Bladder carcinoma	3	4b
21	Bladder carcinoma	3	3b
22	Bladder carcinoma	3	2
23	Bladder carcinoma	3	4a
24	Bladder carcinoma	3	3b
25	Bladder carcinoma	2	Tis
26	Bladder carcinoma	2	Tis
27	Bladder carcinoma	3	3a
28	Bladder carcinoma	3	3b
29	Renal cell carcinoma	3	3a
30	Renal cell carcinoma	2	3a
31	Renal cell carcinoma	3	2
32	Renal cell carcinoma	4	2
33	Renal cell carcinoma	4	3a
34	Renal cell carcinoma	3	1
35	Renal cell carcinoma	4	3a
36	Renal cell carcinoma	3	1
37	Renal cell carcinoma	3	1
38	Renal cell carcinoma	2	2b
39	Renal cell carcinoma	3	3
40	Renal cell carcinoma	3	1a
41	Renal cell carcinoma	2	2
42	Renal cell carcinoma	3	3a
43	Renal cell carcinoma	2	3
44	Renal cell carcinoma	2	2
45	Renal cell carcinoma	4	3a
46	Renal cell carcinoma	2	1b
47	Renal cell carcinoma	3	3b
48	Renal cell carcinoma	2	1b

To avoid the risk of compromising the diagnostic value of surgically excised material, only small (≈5mm diameter size) tissue specimens were collected, although this choice somewhat limited the possibility to perform a more comprehensive array of immunological analyses and in some cases made it impossible to carry out the whole panel of scheduled tests in each single tissue sample. The study was approved by the local ethics committee; all patients and controls enrolled in the study provided written informed consent.

### Purification of peripheral blood mononuclear cells (PBMC) and tissue lymphocyte infiltrates

PBMC were purified from heparinized blood samples by centrifugation on Ficoll-Hypaque gradient (Biochrom AG, Berlin, Germany) for 30 minutes at 1800 rpm.

Lymphocytes from surgical specimens were purified by filtering minced tissues using a sterile cell strainer (Falcon, BD Biosciences, San Josè CA) and running the collected cells on Ficoll gradient.

### Monoclonal antibodies (mAb) and immunofluorescence analyses

Cell expression of membrane antigens was tested by immunofluorescence analysis performed either on PBMC or on tissue lymphocyte infiltrates (1×10^5^ cells in 100 μl of PBS). Cells were incubated with specific mAbs at 4°C for 30 minutes in the dark. The following mAbs were used: fluorescein isothiocyanate (FITC) conjugated anti-CD45RA (Becton Dickinson, (BD) Biosciences, San Josè CA), phycoerythrin (PE) conjugated anti-CD127 (e-Biosciences, San Diego, CA), Peridinin Chlorophyll Protein Complex-cyanin 5.5 PerCPCy5.5conjugated anti-CD28 (eBioscience), Pe-Cy7 conjugated anti-CD25 (BD), allophycocyanin (APC) conjugated anti-CD39 (BD), allophycocyanin (APC)-cyanin (Cy) 7 conjugated anti-CD3 and eFluor 450 conjugated anti-CD8 (e-Biosciences). Fluorochrome-conjugated isotype matched mAbs were used as controls (Becton Dickinson, BD Biosciences, San Josè CA). Following the staining procedures the cells were analyzed by a FACSCanto II flow cytometer (BD Biosciences) using FACS DIVA software (BD Biosciences).

### Intracellular staining

The intracellular production of IFNγ and of IL17A by T lymphocytes from PBMC and tissue infiltrates was analyzed as follows: the cells (resuspended in culture medium conditioned with 10% autologous plasma at a concentration of 1 × 10^7^/ml) were stimulated with phorbol-12-myristate-13-acetate (PMA 50 ng/ml, Sigma) and ionomycin (2 μg/ml, Sigma) for 5 hours at 37°C. Brefeldin A (BFA 10 μg/ml, Sigma) was added to the cells for the last 4 hours of incubation. After washings, the samples were stained with fluorochrome-conjugated mAbs specific for surface markers [PerCPCy5.5 anti-CD3 (BD), APCCy7 anti-CD8 (e-Biosciences) and Violet Live/Dead Fixable Dead Cell stain (Life Technologies, CA, USA)], before fixing and permeabilizing the lymphocytes with the Cytofix/Cytoperm kit (BD Bioscience) following the manufacturer's instructions. The cells were washed in Perm-Wash buffer (BD Bioscience) and incubated with Pe-Cy7 anti-IFNγ (BD) or FITC anti-IL17A (e-Biosciences) mAbs. Thereafter, the samples were washed in Perm-Wash buffer, fixed with FACS Lysing solution (BD Bioscience) and stored at 4°C. The cytokine profile was analyzed using a FACS Canto II flow cytometer (BD Bioscience) by the FACS DIVA software. The gating strategy for phenotypic analyses is shown in Supplementary Figure 3.

### Expression of MAGE genes in tumor and in non-tumoral bladder or renal tissues

Total RNA was extracted from frozen tissue samples using Omnizol reagent (EuroClone, United Kingdom, UK) according to the manufacturer's instructions. For each sample, RNA was resuspended in 50μl RNAse free water before determining quality and concentration by Thermo Scientific NanoDrop 1000 (Thermo Scientific, Wilmington, MA, USA). All these samples were treated with 6 U DNase I (Promega, Madison, WI, USA) and reverse transcription was carried out on 500 ng of total RNA using the Thermo Scientific RevertAid Reverse Transcriptase (Thermo Scientific, Wilmington, MA, USA). The following oligonucleotide pairs were used (sense and antisense, respectively): MAGE-A1, 5′-CGGCCGAAGGAACCTGACCCAG-3′ and 5′-GCTGGAACCCTCACTGGGTTGCC-3′; MAGE-A2, 5′-AAGTAGGACCCGAGGCACTG-3′ and 5′-GAAGAGGAAGAAGCGGTCTG-3′; GADPH, 5′-GGCATCCTGGGCTACACTGA-3′ and 5′-TGGTGGTCCAGGGGTCTT-3′. Classical RT-PCR amplification was performed by DreamTaq DNA polymerase (Fermentas, Amherst NY, USA). The cycling conditions were 5 min at 96°C, 37 cycles of 45s at 94°C, 45s at 60°C, 1 min at 72°C, with a final extension of 10 min at 72°C. We used normalization with GADPH expression to quantify PCR products before testing MAGE gene expression; PCR products were checked by sequence analysis.

### Histology and immunohistochemistry assessments

For each specimen, a specular sample of neoplastic or normal tissues that had been used to perform immunological and molecular analyses were fixed in 10% buffered formalin and then routinely processed in paraffin.

Four 4 μm thick serial sections were freshly cut from each paraffin block by microtome [29]. One section was stained with hematoxylin and eosin and the other three sections were mounted on SuperFrost Plus slides (Thermo Scientific, Braunschweig, Germany) for immunohistochemistry analysis.

Sections for immunohistochemistry were dried, deparaffinized and rehydrated. Endogenous peroxidase was blocked with 5% H2O2 for 10 minutes. Immunoreactions were performed using the automated BenchMark XT immunostainer® (Ventana Medical Systems, Arizona, USA). Standard heat-based antigen retrieval was performed for 30 minutes (when needed). The iVIEWTM DAB detection kit (Ventana Medical Systems, Arizona, USA), which is a streptavidin-biotin based indirect method, was used. After immunostaining, slides were counterstained with hematoxylin and coverslipped. All reactions were carried out on the same day on consecutive runs. Slides were evaluated simultaneously by three observers (LM, FG, MC) who were blinded to the patient's clinical course.

The following antibodies were used:

- anti-CD3 (SP7 clone, ImmunomarkersVentana, prediluted) for T-lymphocyte evaluation;
- anti-CD4 (SP35 clone, Cell Marque, dilution 1:10) for CD4+ T lymphocyte evaluation;
- anti-CD8 (SP47 clone, Cell Marque, prediluted) for CD8+ T lymphocyte evaluation.

Histology and immunohistochemistry assessments were performed using a Leica DMD108 digital microimaging network instrument equipped with Leica Hi Plan 4X objective.

### Statistical analysis

Statistically significant differences between mean values were analyzed by the Mann-Whitney test for nonparametric values or by the paired t test. Correlations between variables were analyzed by the Spearman correlation test. Statistically significant differences between frequencies were analyzed by Fisher's exact test. Differences were considered statistically significant when P < 0.05. The statistical analyses were performed using the GraphPad Prism 4.0 Software (GraphPad Software, Inc, La Jolla, CA).

## SUPPLEMENTARY MATERIAL FIGURES


