# Supplementary material for: Residual tumor micro-foci and overwhelming regulatory T lymphocyte infiltration are the causes of bladder cancer recurrence
Source: Oncotarget. 2016 Jan 25;7(6):6424–35. doi: 10.18632/oncotarget.7024 (PMC4872724; doi:10.18632/oncotarget.7024)
Supplement: Supplementary file 1 [file oncotarget-07-6424-s001.pdf]

Residual tumor micro-foci and overwhelming regulatory T lymphocyte infiltration are the causes of bladder cancer recurrence

Supplementary Material

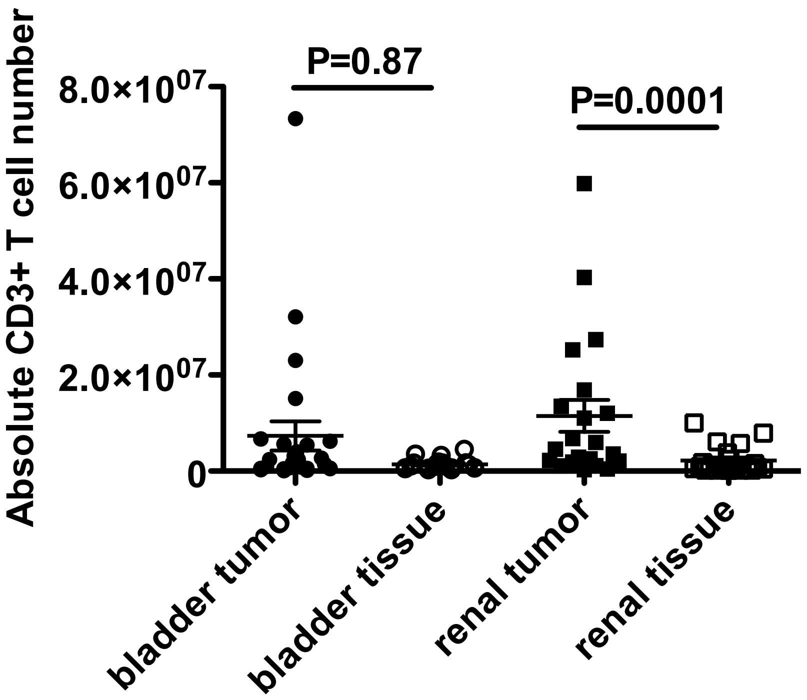

Supplementary Figure 1. Absolute CD3+ T cell counts. The absolute numbers of CD3+ T lymphocytes were normalized for the weight of tissue specimens. Circles: Bladder specimens; squares: renal specimens; black symbols: tumor specimens; open symbols: apparently free-of-tumor bladder or renal tissue.

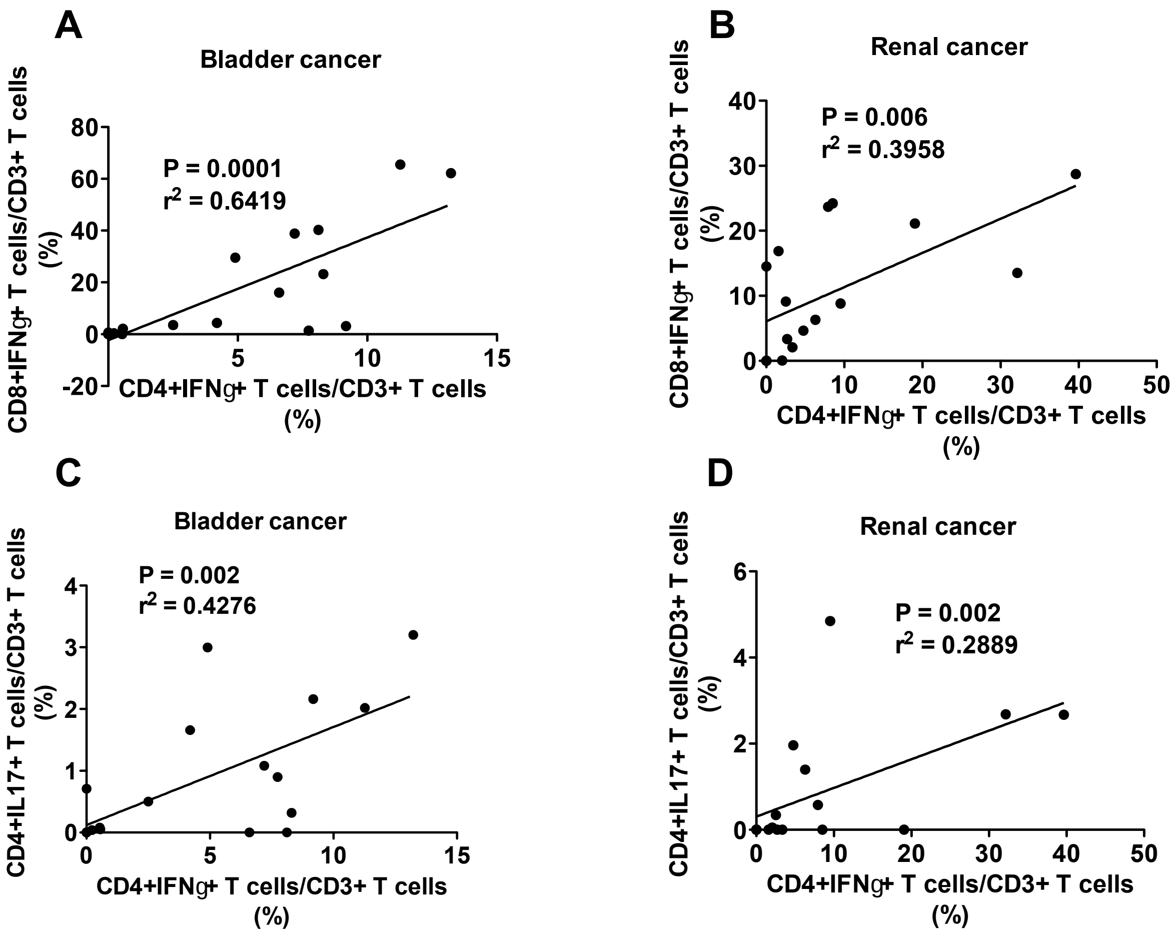

Supplementary Figure 2. Analyses of correlation among frequencies of intratumoral effector T cell subsets. Panels A and C refer to analyses performed on cells from bladder cancer patients; panels B and D refer to analyses performed on cells from renal cancer patients.

A

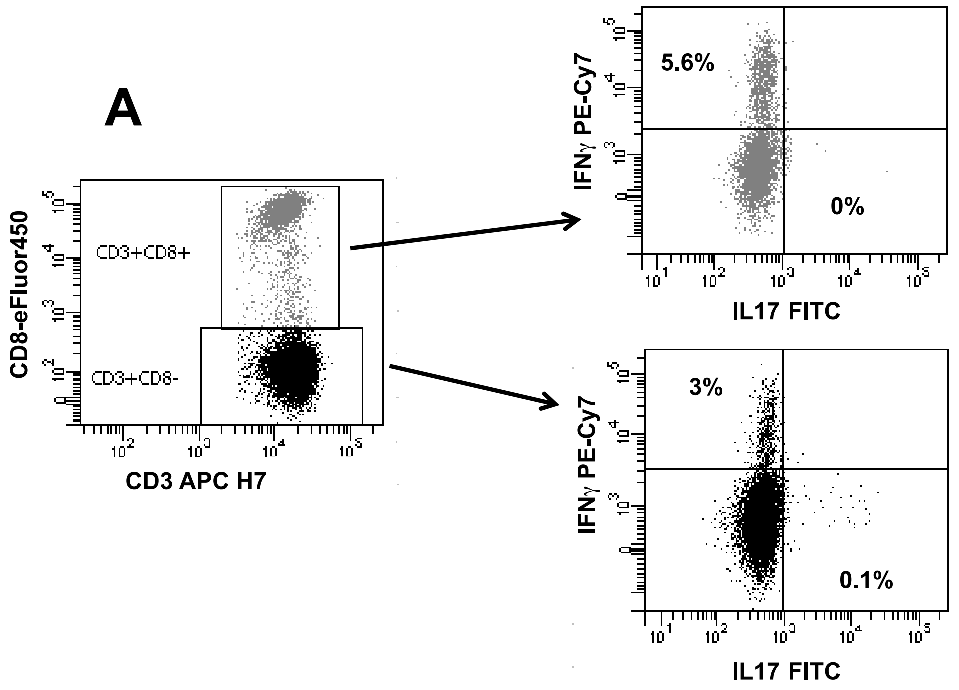
CD3+CD8+
CD3+CD8-
CD8-eFluor450
CD3 APC H7
5.6%
0%
IFNγ PE-Cy7
IL17 FITC
3%
0.1%
IFNγ PE-Cy7
IL17 FITC

B

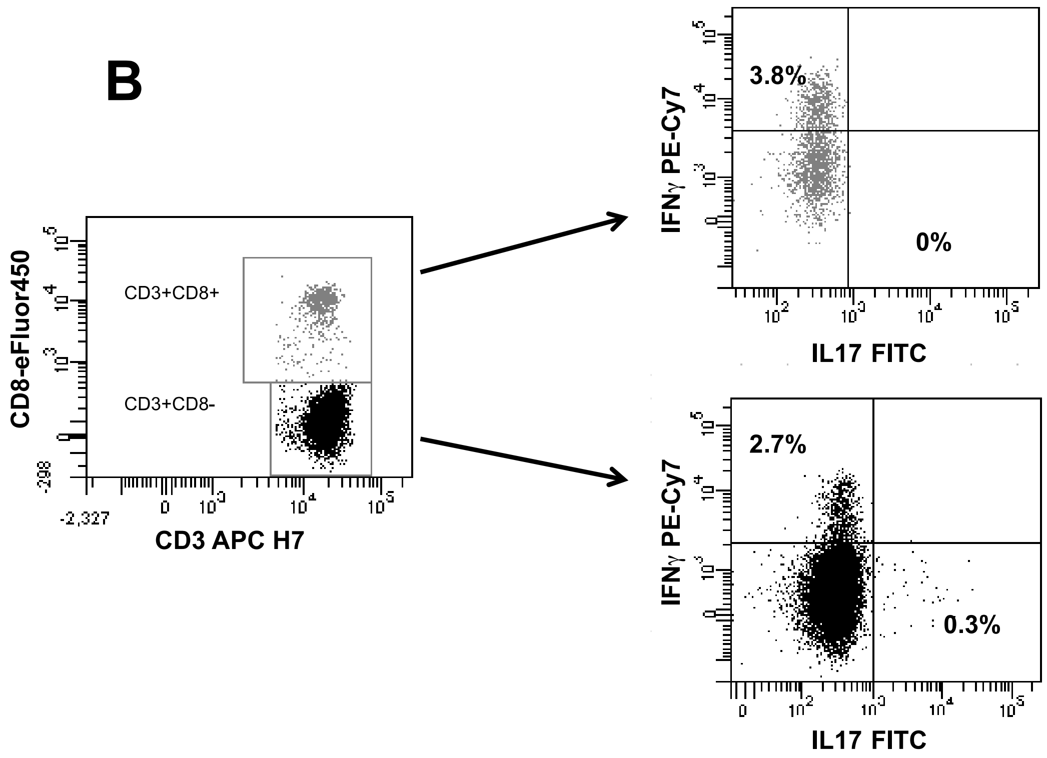
CD3+CD8+
CD3+CD8-
CD8-eFluor450
CD3 APC H7
-298
-2,327
3.8%
0%
IFNγ PE-Cy7
IL17 FITC
2.7%
0.3%
IFNγ PE-Cy7
IL17 FITC

C

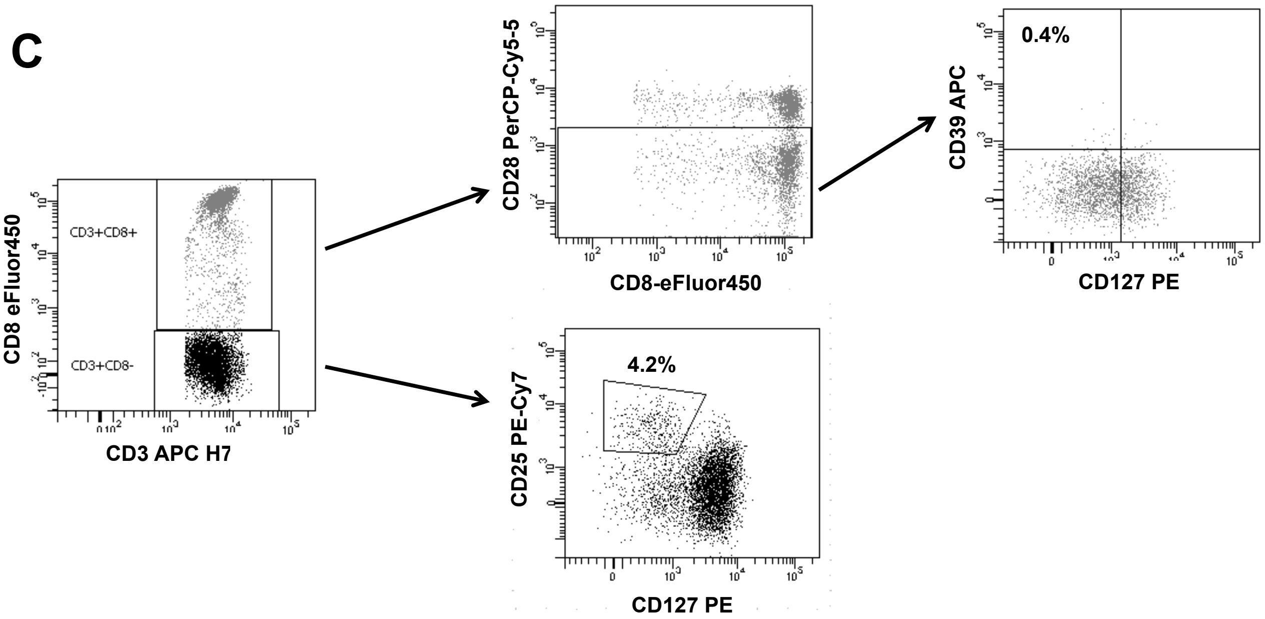
CD8 eFluor450
CD3+CD8+
CD3+CD8-
CD3 APC H7
CD28 PerCP-Cy5-5
CD8-eFluor450
CD39 APC
0.4%
CD127 PE
CD25 PE-Cy7
4.2%
CD127 PE

D

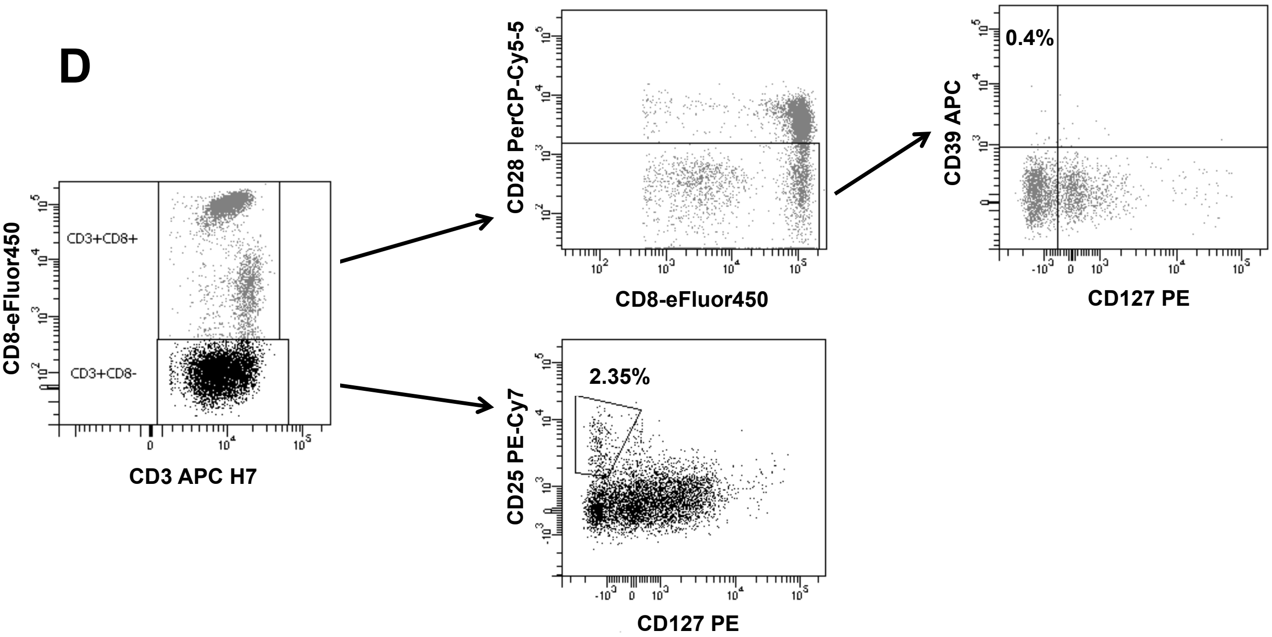
CD8-eFluor450
CD3+CD8+
CD3+CD8-
CD3 APC H7
CD28 PerCP-Cy5-5
CD8-eFluor450
CD39 APC
0.4%
CD127 PE
CD25 PE-Cy7
2.35%
CD127 PE

E

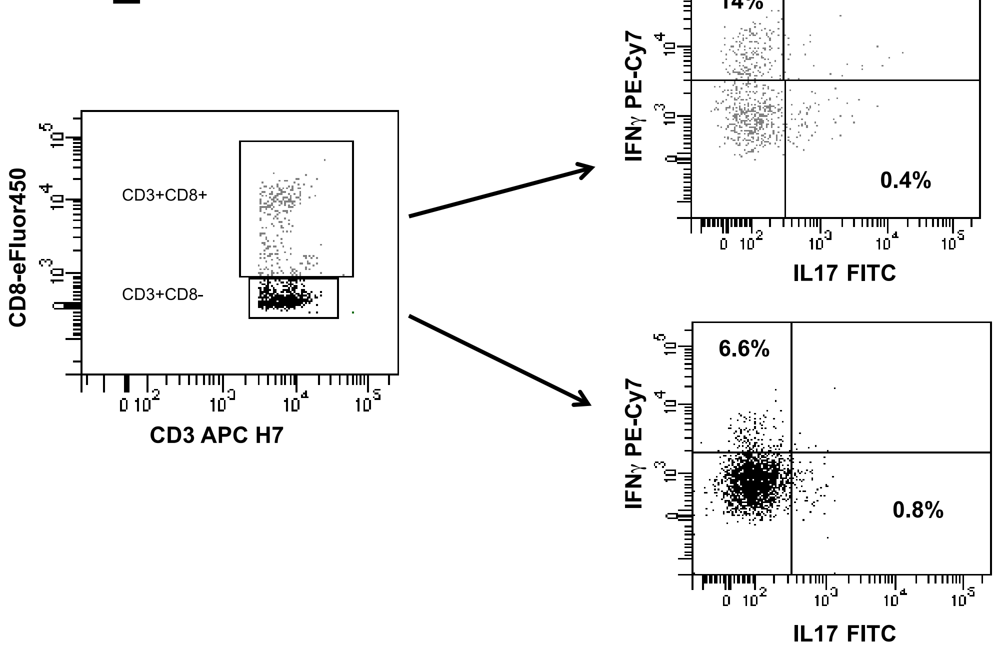
CD8-eFluor450
CD3+CD8+
CD3+CD8-
CD3 APC H7
14%
0.4%
IFNγ PE-Cy7
IL17 FITC
6.6%
0.8%
IFNγ PE-Cy7
IL17 FITC

F

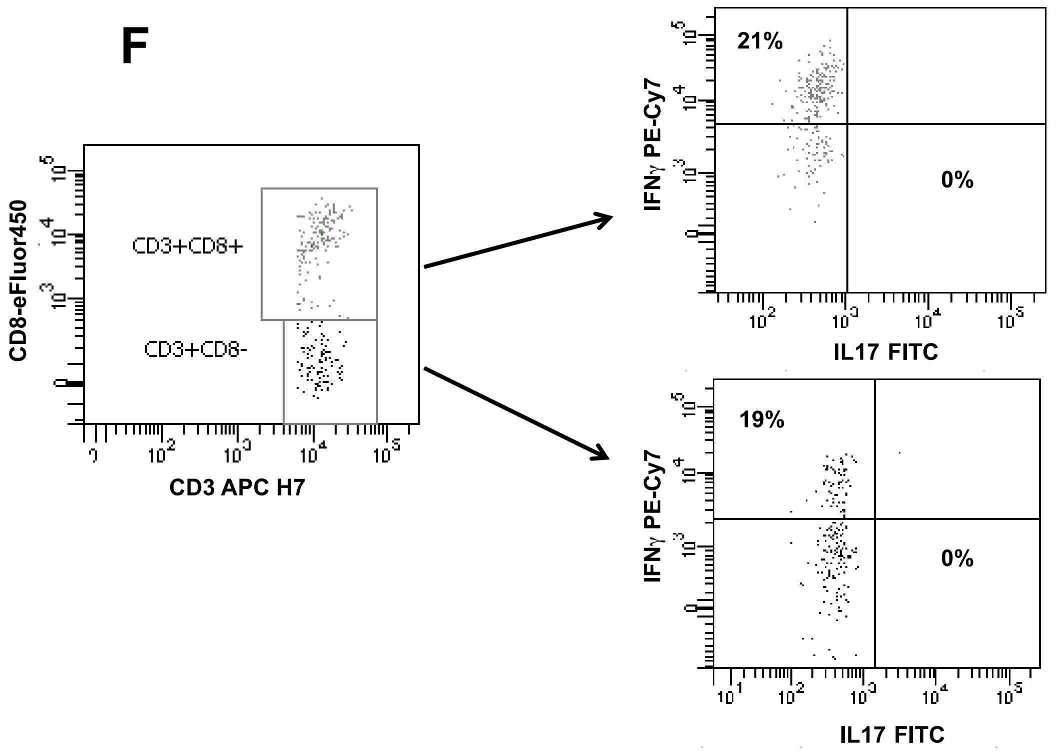
CD8-eFluor450
CD3+CD8+
CD3+CD8-
CD3 APC H7
21%
0%
IFNγ PE-Cy7
IL17 FITC
19%
0%
IFNγ PE-Cy7
IL17 FITC

G

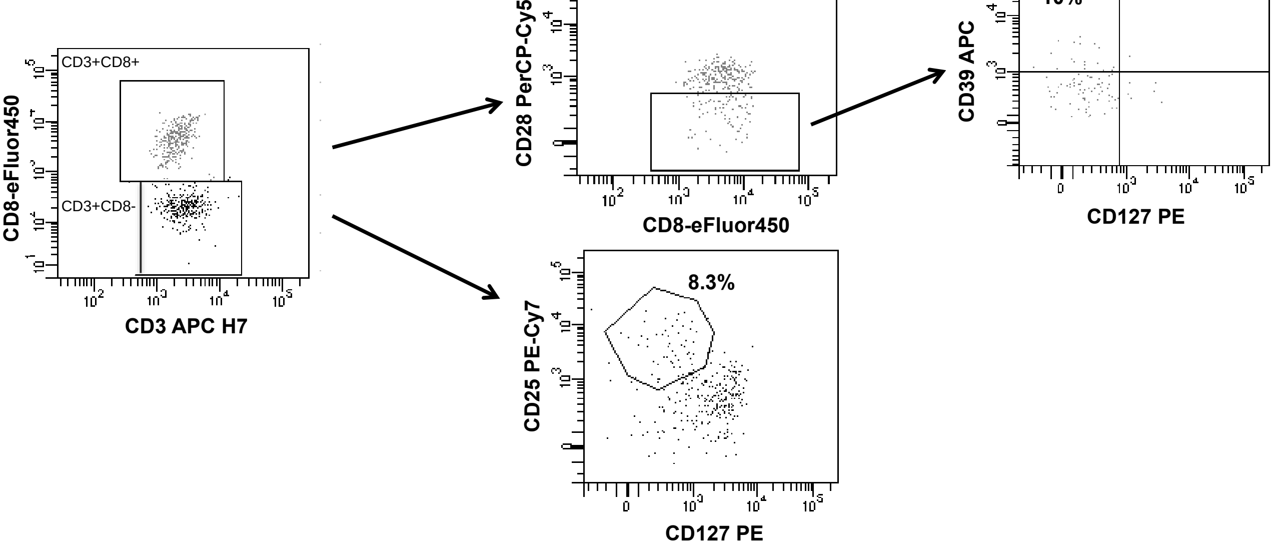

CD3+CD8+
CD3+CD8-
CD8-eFluor450
CD3 APC H7
CD28 PerCP-Cy5-5
CD8-eFluor450
10%
CD39 APC
CD127 PE
8.3%
CD25 PE-Cy7
CD127 PE

H

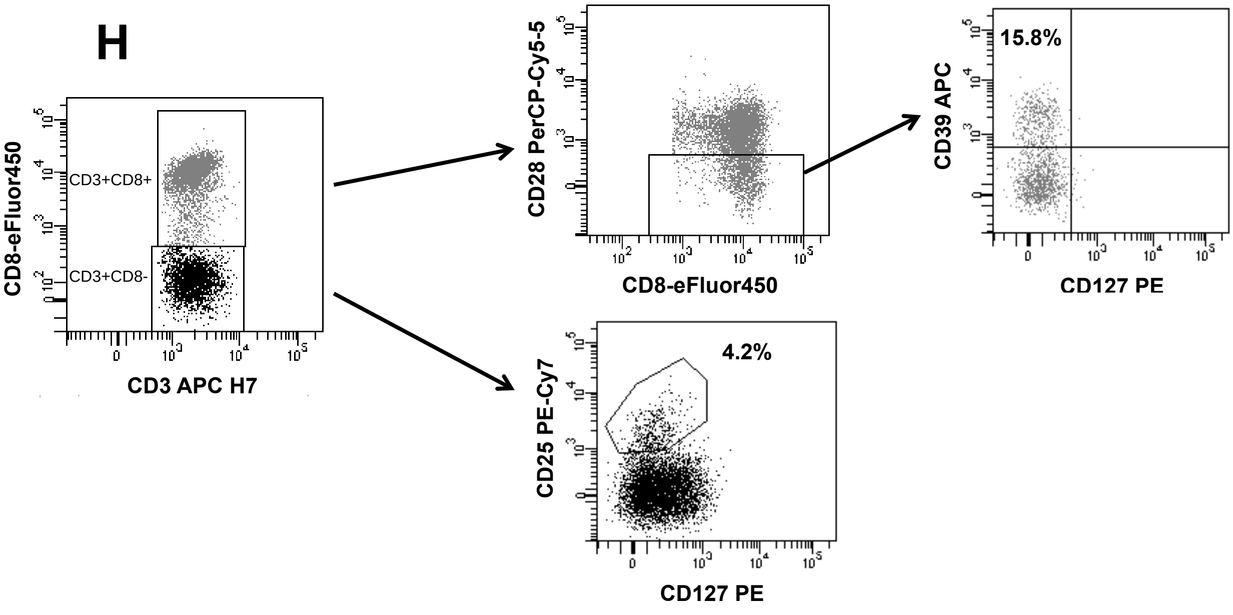

CD3+CD8+
CD3+CD8-
CD8-eFluor450
CD3 APC H7
CD28 PerCP-Cy5-5
CD8-eFluor450
15.8%
CD39 APC
CD127 PE
4.2%
CD25 PE-Cy7
CD127 PE

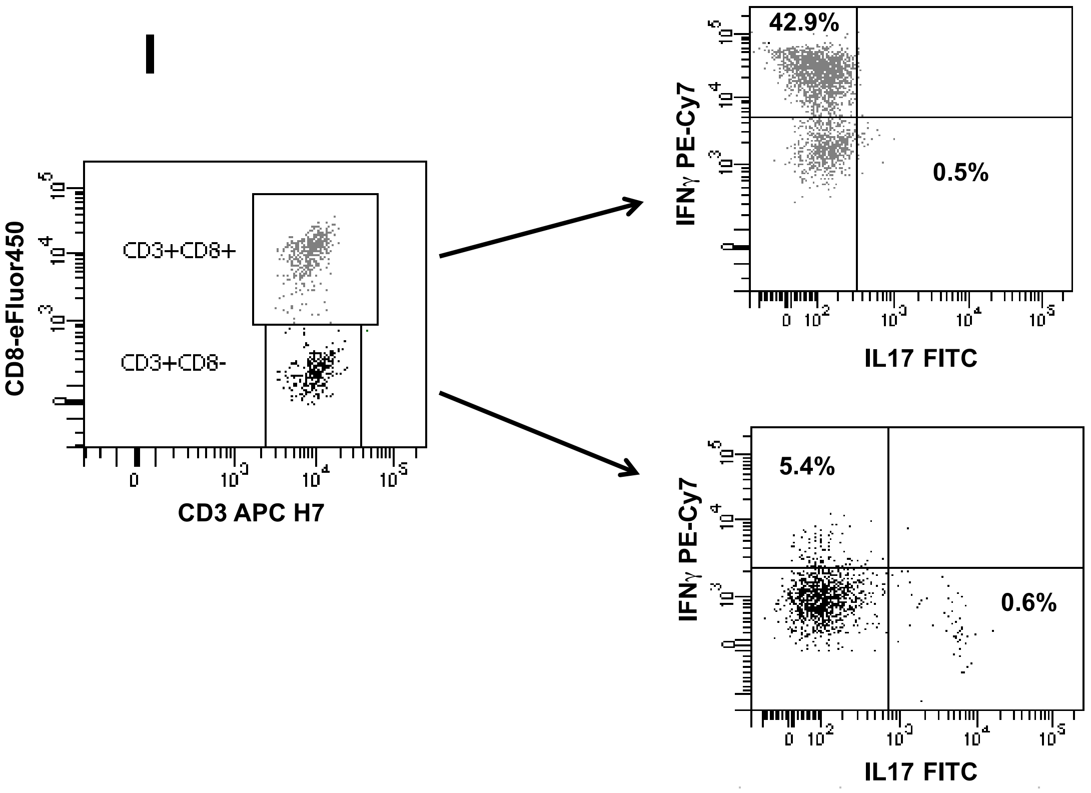
I
CD8-eFluor450
CD3+CD8+
CD3+CD8-
CD3 APC H7
IFNγ PE-Cy7
42.9%
0.5%
IL17 FITC
5.4%
0.6%

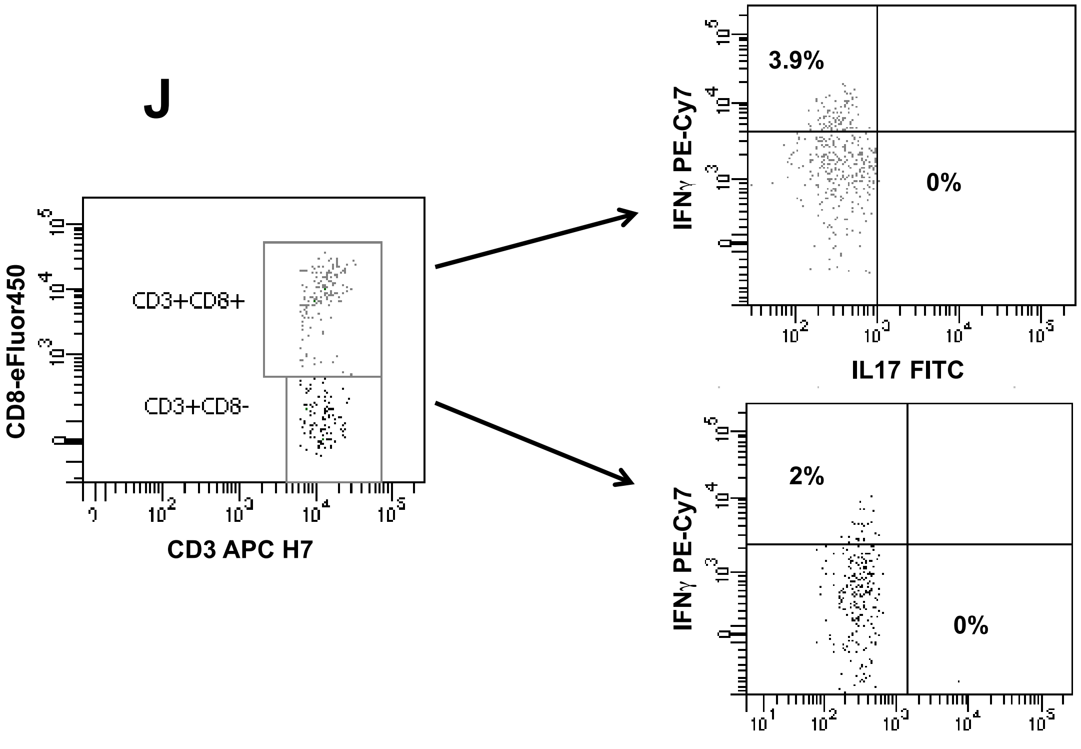
J
CD8-eFluor450
CD3+CD8+
CD3+CD8-
CD3 APC H7
IFNγ PE-Cy7
3.9%
0%
IL17 FITC
2%
0%

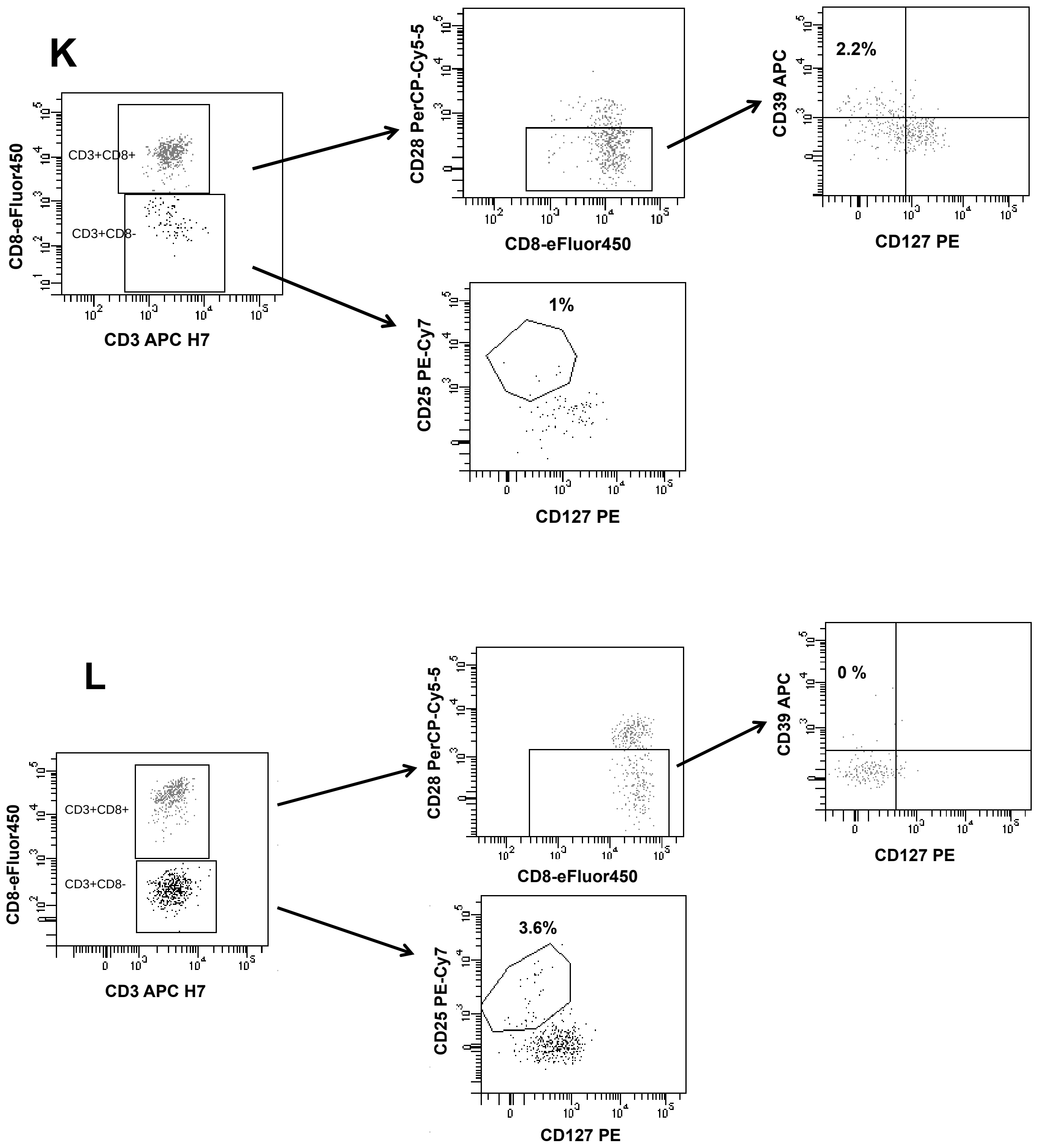

Supplementary Figure 3. Gating strategy for phenotypic analyses. A, C, E, G, I, K: representative analyses performed on T cells from bladder cancer patients; B, D, F, H, J, L: representative analyses performed on T cells from renal cancer patients; A, B, C, D: analyses performed on PBMC; E, F, G, H: analyses performed on tumor infiltrating T cells; I, J, K, L: analyses performed on T cells from apparently free-of-tumor bladder or renal tissues; A and B: analyses performed on effector T cells from PBMC of the bladder cancer patient #24 and the renal cancer patient #45, respectively; C and

D: analyses performed on Treg lymphocytes from PBMC of the bladder cancer patient #19 and the renal cancer patient #23, respectively; E and F: analyses performed on tumor infiltrating effector T cells from the bladder cancer patient #9 and the renal cancer patient #44, respectively; G and H: analyses performed on tumor infiltrating Treg lymphocytes from the bladder cancer patient #13 and the renal cancer patient #39, respectively; I and J: analyses performed on effector T cells from the apparently free-of-tumor bladder (I) or renal (J) tissues of the bladder cancer patient #9 and the renal cancer patient #44, respectively; K and L: analyses performed on Treg lymphocytes from the apparently free-of-tumor bladder (K) or renal (L) tissues of the bladder cancer patient #13 and the renal cancer patient #39, respectively.
